# The Development and Impact of Anxiety With Migraines: A Narrative Review

**DOI:** 10.7759/cureus.26419

**Published:** 2022-06-29

**Authors:** Rajeswar Kumar, Saba Asif, Anoushka Bali, Ashujot Kaur Dang, Daniel A Gonzalez

**Affiliations:** 1 Medicine, Rajah Muthiah Medical College, Chidambaram, IND; 2 Internal Medicine, Apollo Hospitals, Hyderabad, IND; 3 Research, Acharya Shri Chander College Of Medical Sciences & Hospital, Jammu, IND; 4 Research, Government Medical College, Patiala, IND; 5 Medicine, Universidad Catolica Santiago de Guayaquil, Guayaquil, ECU

**Keywords:** chronic migraine (cm), anxiety sensitivity, panic disorders, ocd/ anxiety disorders, generalised anxiety disorder, somatic symptoms of anxiety disorders, migraine headaches, mood and anxiety, migraine-type headache, migraine disorder

## Abstract

Migraine is a chronic, disabling neurological disorder characterized by recurrent episodes of headache. Psychiatric disorders have been reported to arise due to a patient's physical and emotional stress caused by migraine episodes, with anxiety disorders being one of the most commonly associated psychiatric disorder with migraine. This association poses the question of similar or shared pathogenesis between the two disorders and raises a concern for the diagnosis and management of situations when these disorders present together. In this review, we discuss the possible shared mechanism for the development of anxiety disorders in the presence of migraine, such as the vascular, nervous, and genetic factors that might hold the key to their association. We also discuss the number of clinical features shared by these conditions and provide evidence for the higher degree of association between these conditions.

A focused evaluation of anxiety disorders in migraine might benefit patients with earlier diagnoses and improve their quality of life with effective pharmacotherapy and psychotherapy. This review also emphasizes the importance of preventing future migraine episodes with effective prophylactic medications to reduce the risk of developing anxiety disorders, and the need to discuss the medical and psychiatric management of anxiety disorders in patients suffering from migraines on an acute and long-term basis. ​​​​​​

## Introduction and background

Migraine is a common neurological disorder that causes headaches by increased excitability of the neurons of the central nervous system (CNS) [1]. The knowledge of the causes and treatment of migraine is recent even though its presence has been noted since the time of the Ancient Greeks. The term migraine is derived from the Greek word *hemicrania*, which means one-sided headache [2]. In 1870, the disease was first described by the English physician Hubert Airy as a condition characterized by visual auras and 'oppressive headaches', and referred to it as "transient teichopsia" [3]. Later in the 20th century, several studies and hypotheses were made regarding the cause and pathogenesis of migraine. In the 21st century, migraine is the third most prevalent medical condition globally, according to the WHO data [3]. Migraine is found to be more prevalent and severe in women than in men by two to three times, and they are found to experience the attacks for a longer duration. Also, the risk of headache recurrence is increased, and they suffer from a more significant disability and require a longer time for recovery than men [4].

Since migraine is more common in women, it is often underdiagnosed in men resulting in suboptimal management, and hence their participation in clinical trials is less [4]. Although migraine is found to be familial, there is no evidence of any identified genetic association with a large effect size [5]. The significant risk factors for chronic migraine are non-modifiable, and they include age, female sex, and low educational status. However, other factors like obesity, depression, overuse of migraine medications, ineffective treatment of acute episodes, and stressful life events also increase the risk of chronic migraine [6].

The various symptoms of migraine attacks are photophobia, phonophobia, dizziness, nausea, or anorexia; this reflects the complex pathogenesis and diffuse involvement of various neural networks and anatomical regions within the CNS [7]. These symptoms commonly precede the onset of headache in migraine by several hours and are referred to as 'aura'. However, migraine aura is not necessary or sufficient to cause headache since the aura symptoms most commonly occur without a headache, and most migraine attacks do not include any aura [5].

The pathophysiology of migraine, which was once thought to be purely vascular, is now transitioned to a neurovascular hypothesis [8]. A wave of depolarization and suppression of the neurons and glia of the CNS results in alteration of the local blood flow and metabolism in the cortex and the brainstem; this phenomenon is called cortical spreading depolarization (CSD) [9]. The physiological mechanism of a migraine visual aura is thought to be due to CSD [9]. As acknowledged by a PET study, the earliest stage of a migraine attack showed activation of neurons in the posterior hypothalamus and the lateral hypothalamus, and the adjacent midbrain ventral tegmentum [7]. Based on the findings, it was proposed that a brain stem generator initiates the migraine attacks either through an abnormal activation or through a failure of inhibition [10]. The involved areas, such as the hypothalamus and the midbrain, have a central connection with the limbic system, which might explain the alterations in homeostasis and aura symptoms like mood changes [7]. Among the mood and affect disorders, anxiety was found to be more robustly associated with chronic migraine. The patient's inability to control worrying and relax is the major issue with chronic migraine [11].

This review article highlights the association between migraine and anxiety by discussing the similarities in anatomical regions and physiological mechanisms involved in the pathophysiology of these diseases, and how patients with migraines suffer from constant anxiety that occurs as a consequence of the condition's disabling symptoms.

## Review

Migraine and anxiety: A shared pathophysiology

Migraine and anxiety are very closely related due to their similar clinical features, presentation methods, and the episodic nature of their attacks. Anxiety disorders are far more prevalent among migraine patients than in the general population, especially those with chronic migraines [12]. Migraine and anxiety disorders have a bidirectional relationship, with one increasing the risk of the other, although in most cases, it is the anxiety disorder that precedes migraine [13]. In their lifetime, more than 50% of the patients diagnosed with migraine meet the criteria for at least one of the anxiety disorders [14]. The anxiety disorders most commonly associated with migraine are panic disorder (PD), generalized anxiety disorder (GAD), and obsessive-compulsive disorder (OCD) [13].

**Figure 1:**
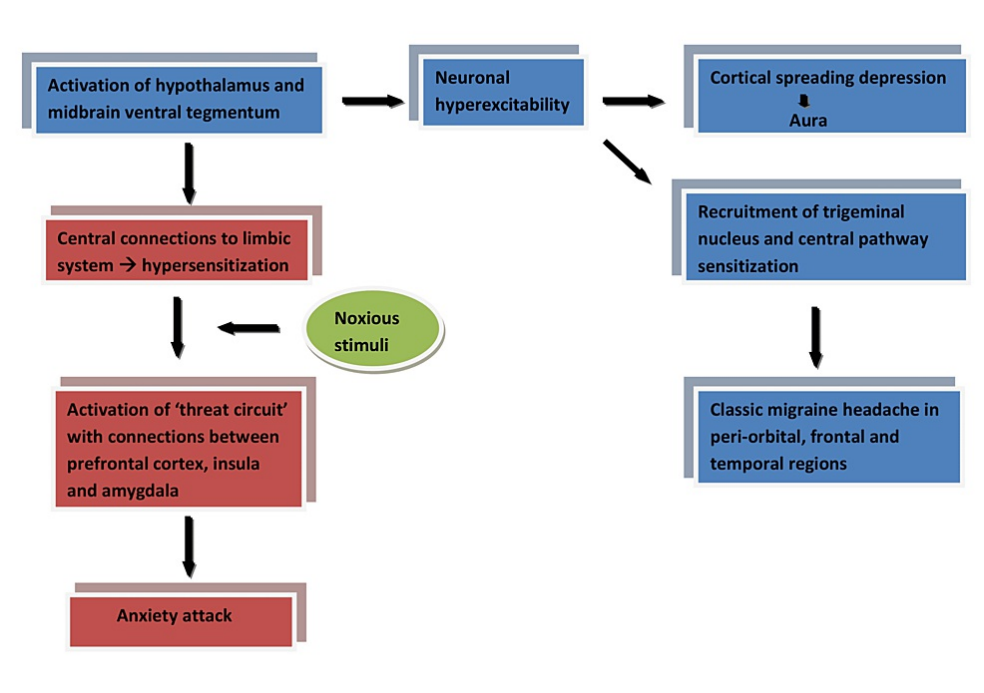
Summary of the shared pathophysiology between migraine and anxiety disorders Image credit: Author Rajeswar Kumar

McWilliams et al. conducted a study that was published in 2004 in the United States among 3032 people from a general population of adults between the ages of 25 to 74 for one year and concluded that there was a higher association of anxiety in patients with migraine (odds ratio of 1.48 to 3.86) [15]. This result might stem from the fact that the pathological mechanisms and the anatomical regions involved in both these conditions are very similar or closely related. The pathogenesis of migraine is believed to be due to neuronal hyperexcitability that leads to CSD, which in turn causes aura; then, the trigeminal nucleus is subsequently recruited, leading to sensitization of the central pathway and pain [16] (Figure 1). The trigeminal nerve's ophthalmic division innervates several pain-sensitive intracranial structures, including the eye, dura mater, large vessels, and dural venous sinuses, which accounts for the classic distribution of migraine headaches involving the peri-orbital, frontal and temporal regions of the head [7]. A premonitory phase or a prodrome of migraine is different from the aura phase in that a prodrome typically lasts longer than a minute and is typically characterized by behavioral features, while the aura phase lasts less than a minute and has neurological features. The pathogenesis of the premonitory phase involves the activation of the hypothalamus and the midbrain ventral tegmentum, which are centrally connected to the limbic system [7] (Figure 1). Current evidence for the pathogenesis of anxiety disorders involves the activation of a 'threat circuit' in the brain with the perception of noxious stimuli that consists of connections between the prefrontal cortex, insula, and amygdala [17] (Figure 1). These structures are a part of the limbic system, and their overstimulation leads to the features of anxiety disorders. Therefore, in a patient with chronic migraine, the limbic system structures are highly sensitized due to the neuronal hyperexcitability that occurs in the regional and neighboring areas in the brain. This sensitization might be the reason for this provocation of anxiety disorders when encountered with a noxious stimulus. Also, the two aspects of anxiety i.e., state and trait have also been studied and co-related with anatomical changes; the anterior cingulated cortex and amygdala were found to be associated structurally with trait anxiety and functionally with state anxiety. The changes in the surrounding environment are detected by the cingulo-opercular network which then connects the anterior cingulate gyrus and insula to the emotional relay node of the amygdala [18].

There are also other hypotheses for the association of migraine with anxiety disorders. One possible mechanism is the serotonergic pathway dysfunction which is hypothesized to be one of the mechanisms for migraine pathogenesis as well as that of anxiety disorders [19-22]. There is found to be an altered metabolism of serotonin (5-HT) in patients with serotonin transporter gene polymorphism and is seen both in patients with migraine and those with anxiety disorders [20]. There is also evidence that the 5-HT1 receptor agonists (triptans) are effective in treating migraine [19]. In contrast, the serotonin pathway has long been implicated in the pathogenesis of anxiety disorders, with evidence that selective serotonin reuptake inhibitors (SSRI) are effectively used in anxiety disorders [23]. However, in contrast to this assumption, a study by Karwautz et al. conducted in 2007 on two functional 5-HT transporter gene polymorphisms in patients with migraine revealed no significant association between the presence of the haplotypes and susceptibility to migraine [24].

Another hypothesis is that the psychological factors in chronic headache conditions such as migraine might play an essential role in the development of anxiety disorders and even lead to the chronicity of migraine [23]. One such factor is anxiety sensitivity (AS) in patients with chronic headache disorders. This factor alone, even without the presence of other psychological factors, might contribute to the development of anxiety disorders in chronic headache patients [25]. Anxiety sensitivity can be explained as increased sensitivity to the fear of pain and pain-related experiences and activities; this later leads to the tendency of patients to be fearful of those situations even when they are not associated with a stimulus for pain [26]. With repeated attacks of migraine headaches, the patients develop a catastrophic misinterpretation of physical sensations in negative ways [23]. For example, they might misinterpret normal sensations for signs of severe illness like an impending heart attack. Such misinterpretation leads to an increased sensitivity to the fear of pain, and the patients develop avoidance behaviors of those physical sensations [23]. Pain-related anxiety is a distinct terminology from AS. It differs from AS in the sense that it is the fear of inherently noxious stimuli without their misinterpretation in negative ways, whereas in AS, normal physical stimuli are misinterpreted as highly noxious [27]. Norton et al. conducted a study in 2004 using structural equation modeling among patients experiencing recurrent headaches; the results of the study concluded that pain-related fear, escape, and avoidance behavior are greatly affected by AS in patients with recurrent headaches [28].

**Figure 2:**
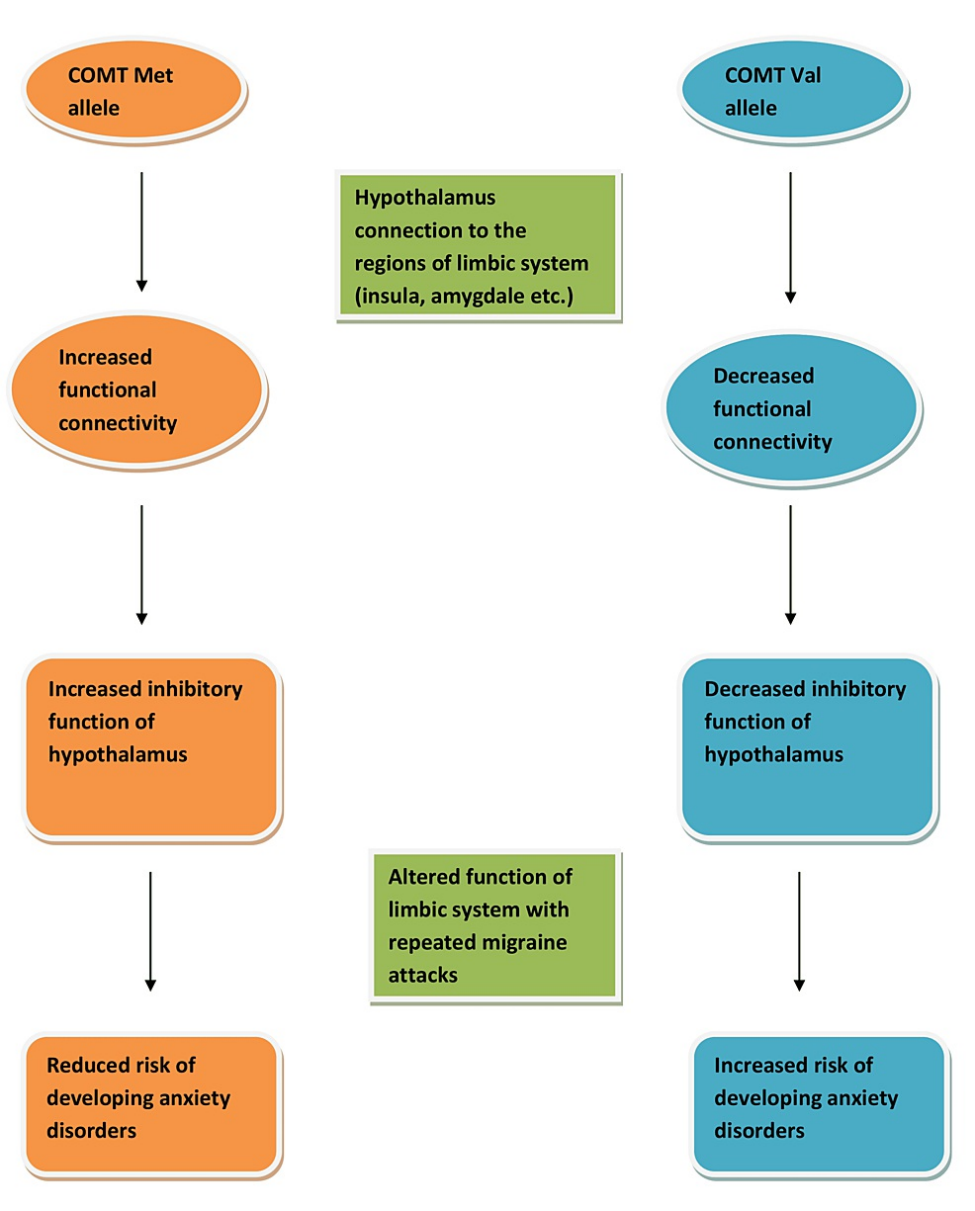
Genetic association for the development of anxiety disorders in migraine patients COMT Met allele: Catechol-o-methyl transferase methionine allele, COMT Val allele: Catechol-o-methyl transferase valine allele Image credit: Author Rajeswar Kumar

Certain genetic factors have also been hypothesized to be involved in the association of anxiety disorders with migraine. In patients with chronic migraine that suffer from repeated migraine attacks, it is found that there is an alteration in the architecture and function of several regions of the limbic system and also an alteration in the functional connectivity of the hippocampus with other brain regions [29]. These regions include the insula, amygdala, cingulate cortex, caudate, putamen, and prefrontal cortex [29]. A genetic association was found to be related to the varying functional connectivity of the hippocampus with these regions. Patients with the catechol-o-methyl transferase methionine allele (COMT Met allele) were found to have higher functional connectivity, whereas the patients with homozygous catechol-o-methyl transferase valine allele (COMT Val allele) were found to have lower functional connectivity [30] (Figure 2). This alteration in the functional connectivity of the hypothalamus is responsible for the altered architecture and function of the limbic system, which in turn is the reason for the state of anxiety [30]. The hypothalamus has an inhibitory function over these regions; therefore, the patients with the COMT Met allele are at a lower risk for developing anxiety disorders with repeated migraine attacks, whereas patients with the homozygous COMT Val allele are at an increased risk of suffering from anxiety disorders from repeated migraine attacks (Figure 2).

Migraine and anxiety: Clinical features and associations

The clinical features of migraine include unilateral, throbbing, or pulsating headache episodes, most commonly over the temple. The headache might or might not be associated with an aura, characterized by periodic attacks and a return to baseline between the episodes [16]. The episodes of migraine headaches are often associated with autonomic disturbances and are accompanied by symptoms like nausea and vomiting, and very rarely might lead to seizures [31]. Anxiety is a state of mood which is future-oriented and is associated with the preparation for possible, upcoming adverse outcomes [32]. Psychiatric disorders with anxiety as a major component include GAD, PD, OCD, post-traumatic stress disorder (PTSD), acute stress disorder (ASD), social phobia, and specific phobia [13]. Both GAD and PD are the most common anxiety disorders. Panic attacks are characteristic of PD and are characterized by sudden, unexpected, extreme episodes of mental and biological fearfulness and the return to baseline after some time [13]. The features of GAD include chronic, pervasive worry and anxiety associated with other nonspecific symptoms that may be physical or psychological, such as fatigue, lack of concentration, and sleep disturbances [33]. Other anxiety disorders are specific to certain circumstances and age groups. For instance, PTSD is most common after an incident with a strong emotional impact, and separation anxiety disorder is most common in children under the age of 10 years.

Migraine and anxiety disorders share several standard clinical features that may result in delay and difficulty in diagnosing the development of anxiety disorders in migraine. Some of the shared clinical features between migraine and anxiety disorders are that both the conditions manifest as paroxysmal, brief episodes with a return to baseline after the attack. They can be precipitated by certain physical, emotional, or social stimuli and associated with a tendency for behavioral avoidance of such stimuli. Psychosocial stress is the most common trigger for migraine attacks [34], the same as anxiety disorders. Both the conditions are associated with an autonomic abnormality that manifests as a feature of the disease (e.g., nausea and vomiting in migraine; sweating and palpitations in PD). Both migraine and PD are associated with similar psychological symptoms, such as AS and chronic worry [31]. Rarely, both the conditions may lead to mental confusion or a decrease in the level of consciousness during the attacks.

Panic disorder especially shares several clinical features with migraine, such as autonomic disturbances, functional impairment, gastrointestinal disturbances, and fear and worry symptoms between episodes [23]. The development of PD in patients with migraine has a poorer prognosis because it also leads to increased disability, increased risk of medication overuse, and worsening the frequency and duration of the headache episodes [23]. 

Significant overlap is evident between the two disorders' clinical features, which in itself suggests the possibility of common pathophysiology. It also indicates a higher possibility for the co-occurrence of both these disorders. Also, the presence of anxiety disorders in patients with migraine significantly affects the patient's quality of life. A cohort study in 2004 by Pesa et al. revealed that migraine patients with co-morbid anxiety had very high medical costs and expenses compared to migraine patients without any co-morbidity [35]. This study indirectly reflects how the quality of life is affected in those patients.

In a study by Sharma et al. in 2013, patients newly diagnosed with migraine were assessed for several predictors of quality of life such as Short Form-36 (SF-36), migraine disability assessment score (MIDAS), and hospital anxiety and depression scale [36]. The study revealed that the presence of clinically significant anxiety and its severity were major factors that led to significant impairment in all the predictors used in the assessment of the quality of life [36]. Therefore, it becomes imperative to manage and control anxiety disorders in patients with migraine. Controlling the anxiety in patients with migraine would lead to an improved quality of life and better and more effective treatment for migraine headaches [13].

Several studies have been made on the association of anxiety disorders in patients with migraine. Unsurprisingly, most studies reveal an increased incidence of anxiety disorders in patients suffering from chronic migraine [37-40]. The association is more evident in patients with chronic migraine since the development of anxiety disorders is linked to repeated migraine headaches that occur over a certain period. One such study by Lantéri-Minet et al. was a population-based postal survey study conducted in 2005 in France among 1,957 patients with active migraine who were compared to 8,287 subjects without migraine; the results of the study indicated a significant association between anxiety in active migraine with more than 50% of the patients with active migraine suffering from anxiety [37].

A cohort study conducted by Merikangas et al. in 1990 in Zurich, Switzerland, among subjects aged 27 and 28 in a general population with a migraine prevalence of 13.3%, demonstrated a significant association between migraine and anxiety and an even stronger association between a combination of anxiety and major depression with migraine [38]. These kinds of studies further emphasize the association and co-presentation of migraine and anxiety disorders and a possible shared underlying pathology. Whereas another study by Beghi et al. conducted in 2007 in Italy among 374 adult migraine patients from secondary and tertiary care centers was designed with adult migraine patients without aura as the case group and two control groups including patients with pure tension-type headache, and patients with combined migraine and tension-type headache. The conclusion of the study reports that anxiety disorders were shared among all three groups and concludes that the psychopathology of the headache disorders might not be characteristic of a particular headache disorder but instead can be a reflection of the burden of the disease [39].

Nevertheless, in another study in 2002, Jette et al. conducted a population-based study in Canada among 36,984 subjects using a National health survey. They concluded that migraine and anxiety together led to poorer health outcomes when compared to either disease alone [40]. This study reflects the need for specific importance to be given to psychiatric co-morbidities such as anxiety in migraine and signifies the necessity for effective management strategies.

A summary of the above-mentioned studies examining the association of anxiety disorders with migraine is listed below in Table 1.

**Table 1:** Summary of the included studies examining the association of anxiety disorders with migraine

References	Year	Design	Population	Method	Comments
Beghi et al. [39]	2007	_	Adult patients in Italy from secondary and tertiary centers	N=374 patients with pure migraine without aura compared to patients with pure tension-type headache and patients with both	Anxiety was associated with all three groups and no significant difference was noted between them. The psychopathology is not characteristic of a headache disorder but rather is a reflection of the burden of the disease.
Lantéri-Minet et al. [37]	2005	Population-based study	The general population in France	N=1957 patients with active migraine compared to N=8287 non-migraine subjects	A significant association between anxiety in active migraine was found in more than 50% of patients with active migraine suffering from anxiety
McWilliams et al. [15]	2004	_	Adults between the ages of 25 to 74 in the United States	N=3032 patients with migraine compared to controls	Patients with migraine had a higher association with anxiety than those without migraine
Jette et al. [40]	2002	Population-based study	The general population in Canada	N=36,984, National health survey	Patients with migraine and anxiety together had poorer outcomes than patients with either disease alone
Merikangas et al. [38]	1990	Cohort study	The general population of Zurich, Switzerland	Subjects between the ages of 27 and 28 with a migraine prevalence of 13.3%	A significant association between migraine and anxiety and an even higher association between a combination of migraine and major depression

Management

Pharmacotherapy for Acute Management

The management of anxiety disorders in patients with migraine involves both pharmacotherapy and psychotherapy. While psychotherapy is specific to the type of anxiety disorder, most anxiety disorders are managed with similar pharmacotherapy. There are five classes of medications currently used for the management of anxiety disorders in migraine patients: selective serotonin reuptake inhibitor (SSRI), serotonin-norepinephrine reuptake inhibitor (SNRI), azapirones, benzodiazepines (BZD), and beta-blockers [17]. All of these medications are rendered safe to use in patients with migraine except BZD due to their potency for abuse and dependence.

The management of anxiety disorders in migraine involves a step-wise approach. The best initial approach is to provide appropriate education and reassurance to patients. The first line of medication includes the SSRI/SNRI, with solid evidence for their efficacy demonstrated by previous studies [41]. These drugs need careful consideration before use in patients with migraine as they may precipitate a severe condition called 'serotonin syndrome' if patients are already on triptans or valproate for migraine prophylaxis [42]. This syndrome is characterized by autonomic hyperactivity, fever, and altered mental status. Medications of the class azapirones have their efficacy limited to specific categories of anxiety disorders; for example, buspirone, an azapirone, is effective only in the management of GAD [17]. Benzodiazepines and beta-blockers are used as short-term agents for managing acute anxiety; for example, alprazolam/clonazepam is used for acute panic attack management, and propranolol is used as a drug for the performance anxiety sub-type [43]. Other agents that are sometimes used for treating anxiety in migraine patients are tricyclic anti-depressants (TCA), monoamine oxidase inhibitors (MOAI), and hydroxyzine [42]. These drugs are generally not required in migraine patients developing anxiety. They are used only as a final resort when the patient does not respond to the above-mentioned drugs or if they are contraindicated.

The SSRI/SNRI are considered the first-line drugs not only for their proven efficacy but also because of fewer anticholinergic side effects and toxicity compared to TCA and MOAI [44]. The only consideration is for the development of serotonin syndrome, so the patients must be evaluated first for medications they were previously prescribed [42]. Benzodiazepines are recommended only for managing conditions like sudden panic attacks in patients with PD, and the long-term use of BZD is not recommended because of their tendency for abuse and dependence, especially in patients with migraine [45]. Tricyclic anti-depressants and MAOIs are reserved and are essentially considered second-line medications because of their potential side effects of antihistaminic and antimuscarinic activity and issues with tolerability [46]. There are several drugs currently being investigated for the management of anxiety disorders. Some of the emerging drugs that might help in treating migraine patients with anxiety are vortioxetine, vilazodone, agomelatine, guanfacine, nepicastat, aripiprazole, pregabalin, ketamine, and others [46].

In addition, dopamine antagonists are sometimes used for the treatment of migraine, especially antipsychotics. The D2-receptor blocking effect of these medications is the reason for their efficacy with migraine and is used as the first-line medication in the emergency room for migraine patients with nausea and vomiting [47]. The antipsychotics are also sometimes used in the pharmacotherapy augmentation strategies for patients with anxiety disorders who fail to respond to first-line medications and have been found to be well-tolerated and effective for short-term treatment [48]. Such usage of antipsychotics for both migraine and anxiety treatment suggests a possible shared underlying pathological mechanism involving the alteration of dopamine pathways in these conditions

For the acute management of anxiety disorders in migraine patients, the above-mentioned pharmacological therapy is recommended. In addition, specific management for migraine is also required if patients co-present with symptoms of both the disorders.

Pharmacotherapy for Long-term Management

The long-term management of anxiety disorders in chronic migraine involves the prevention of future attacks to reduce morbidity and interference with regular daily activities. With studies that have proven the association of anxiety disorders with recurrent headaches from chronic migraine and poorer outcomes with association [37-40], it becomes necessary to mitigate the attacks of future migraine headaches for a substantial reduction of the development of anxiety disorders.

Drugs used to treat anxiety disorders, such as SSRI, have insufficient evidence for efficacy in the prophylactic management of migraine [49]. However, some studies have provided evidence for their use in preventing future migraine episodes. A randomized, double-blind, parallel study conducted by d'Amato et al. in 1999 conducted at the University of Naples, split adults between the age of 18 and 65 who suffered from migraine without aura into two groups [50]. The patients of one group received fluoxetine and the other received a placebo, and the results of the study revealed a significant reduction in the total pain index starting from the third month in the group receiving fluoxetine compared to the placebo group. This study supports the use of SSRI for migraine prophylaxis which would greatly benefit patients with anxiety and migraine since a single drug would treat both the conditions. However, another study conducted in 1999 by Landy et al. among 27 patients with migraine who were randomized to receive either sertraline or placebo over eight weeks revealed that sertraline was not effective in reducing the frequency and severity of migraine when compared with the conventional drugs used for prophylaxis [51]. This study recommends against the use of SSRIs for migraine prophylaxis. However, further studies would be required to establish the effectiveness of SSRIs in patients with migraine. Venlafaxine, an SNRI, is one of the first-line medications used to treat anxiety disorders which has also been considered to be effective for the prophylaxis of migraine. A randomized, prospective study by Ozyalcin et al. conducted in 2005 among 60 patients diagnosed with migraine without aura, revealed that venlafaxine was significantly effective in the prophylaxis of migraine when compared to placebo [52]. Another study by Hedayat et al. was conducted in 2022 among 80 patients with migraine with a meaover a period of eight weeks to compare the efficacy of amitriptyline and venlafaxine in preventing migraine attacks [53]. The results of the study revealed that both the drugs were significantly effective in reducing the number of migraine attacks and that the side effect profile made venlafaxine a better choice than amitriptyline [53]. The studies by Ozyalcin et al. and Hedayat et al. show that venlafaxine, which can be used to treat anxiety disorders in migraine patients, can also potentially prevent further attacks of migraine, which would, in turn, minimize the severity of anxiety.

Beta-blockers are a class of drugs that have already been extensively studied with known efficacy in migraine prophylaxis (especially propranolol) and the immediate management of anxiety (e.g., performance subtype). The efficacy of propranolol is the same as that of BZD for the short-term treatment of PD [54]. However, propranolol is generally preferred due to milder side effect profiles and abuse potential compared to BZD. Although at present, there is not sufficient evidence for recommending the use of propranolol for treating any anxiety disorders [54].

The prevention of anxiety disorders involves selective measures for people with increased risk, such as patients with high AS, avoidance behaviors, inhibition, and a family history of anxiety disorders [55]. Migraine patients tend to have an increased AS, behavioral avoidance, and inhibition. Therefore, patients with migraine should be followed up regularly for the development of anxiety disorders and treated with long-term management strategies for the condition. 

Psychotherapy

Long-term management specific for anxiety disorders in migraine is generally non-pharmacological and involves psychotherapy. Cognitive-behavioral therapy (CBT) is the widely accepted and most effective long-term management strategy for anxiety disorders [43]. Cognitive-behavioral therapy is one type of psychotherapy that involves attempting to change the thoughts and behaviors of the patient that pertain to maintaining a state of anxiety [17]. The goal of the CBT is to identify and challenge the cognitive biases of the patient that tend to overestimate the risk of a situation and lead to a sense of their inability to manage the risk, and to reduce the behavioral avoidance that manifests as a result of anxiety [17]. Recently, group-based CBT programs have been studied and have shown to provide positive reinforcement strategies, social support, and opportunities for peer modeling, especially in children [56].

In pediatric populations, CBT has shown efficacy even for the management of migraine. A meta-analysis by Ng et al. in 2016 of 3841 articles from the Pubmed and Ovid database revealed the presence of significant evidence that CBT is beneficial in pediatric migraine and may even augment the efficacy of amitriptyline [57].

Even in adult populations, group-based CBT has provided access to better evidence-based treatment and increased clinical efficacy for anxiety disorders [54]. Cognitive-behavioral therapy for anxiety disorders in adult populations may also be effective in migraine prophylaxis. A randomized controlled trial by Richardson et al. in 1989 among 48 migraine patients between the ages of 18 and 50 over six months revealed a significant reduction in the frequency and severity of the headache attacks. Furthermore, the cost-effective minimal contact approach was found to be as effective as the clinic-based approach [58]. Therefore, CBT, which is the primary psychotherapy for anxiety disorders, can also be effective for the management and prophylaxis of migraine. Cognitive-behavioral therapy for patients with migraine who develop anxiety disorders would be incredibly beneficial by increasing the duration of symptom-free periods and might aid in improving their quality of life.

A summary of the above-mentioned studies examining the efficacy of pharmacotherapy and psychotherapy to treat anxiety disorders in patients with migraine is listed below in Table 2.

**Table 2:** Summary of the included studies examining the efficacy of pharmacotherapy and psychotherapy to treat anxiety disorders in patients with migraine CBT: Cognitive behavioral therapy

References	Year	Design	Population	Method	Comments
Hedayat et al. [53]	2022	_	Patients with a mean age of 33	N=80 patients with migraine compared for the efficacy of venlafaxine and amitriptyline in preventing migraine attacks	Both drugs significantly reduced the number of migraine attacks. Venlafaxine is a better choice than amitriptyline due to its side effect profiles.
Ng et al. [57]	2016	Meta-analysis	_	Review of 3841 articles from Pubmed and Ovid database	Significant evidence to prove that CBT is beneficial in pediatric migraine and may even augment the efficacy of amitriptyline
Ozyalcin et al. [52]	2005	Randomized, prospective study	_	N=60 patients with migraine without aura comparing venlafaxine in the prophylaxis of migraine to a placebo	Venlafaxine was significantly effective in the prophylaxis of migraine when compared to a placebo
d’Amato et al. [50]	1999	Randomized, double-blind, parallel study	Adults between the age of 18 and 65 at the University of Naples	Patients who suffered from migraine without aura were separated into two groups with one group receiving fluoxetine and the other receiving placebo	Significant reduction in the total pain index starting from the third month in the fluoxetine group compared to the placebo group
Landy et al. [51]	1999	_	_	N=27 patients with migraine who were randomized to receive either sertraline or placebo over eight weeks	Sertraline was not effective in reducing the frequency and severity of migraine when compared with the conventional drugs used for prophylaxis
Richardson et al. [58]	1989	Randomized controlled trial	Adults between the ages of 18 and 50	N=48 migraine patients treated with CBT over six months	Significant reduction in the frequency and severity of the headache attacks

Limitations

This study does not consider and take into account patients with particular conditions suffering from migraine (e.g., pregnancy, depression) and other environmental variables that might affect the susceptibility to anxiety disorders in patients with migraine. This study also does not consider the impact of the COVID-19 pandemic on patients with migraine and anxiety disorders and also does not consider the development of migraine headaches or anxiety as a complication of COVID-19. Also, in the evaluation of the efficacy of treatment, the study did not consider the specific type of anxiety disorder or the severity of the disease at the time of presentation or in the trials. 

## Conclusions

Patients with migraine are very susceptible to anxiety disorders, as evidenced by the studies reviewed in this article. The relationship between anxiety disorders and migraine headaches appear to be bidirectional as recurrent headaches in migraine lead to anxiety and vice-versa. The reason for the association may be due to underlying unknown pathogenesis. Both these disorders share several standard features making it difficult to diagnose the development of one disorder in the presence of the other. Several studies have confirmed the association of anxiety disorders with migraine, and the co-presentation of these disorders significantly affect the quality of life in such patients. In light of these factors, it becomes crucial to screen for the presence or development of anxiety disorders in patients with migraine and the need to manage these conditions effectively. Preventing the development of anxiety disorders in migraine patients is the best strategy and is achieved by minimizing the number of headache episodes using effective prophylactic pharmacotherapy. For patients who develop anxiety disorders, SSRIs are the first-line pharmacotherapy. As for psychotherapy, studies have shown the significance of CBT in effectively managing anxiety disorders. Finally, we recommend that more significant studies be performed on the relationship between migraine and anxiety disorders for a more coordinated and direct approach to aid in the diagnosis and management of these conditions.
